# Comment on Sycinska-Dziarnowska et al. The Implications of the COVID-19 Pandemic on the Interest in Orthodontic Treatment and Perspectives for the Future. Real-Time Surveillance Using Google Trends. *Int. J. Environ. Res. Public Health* 2021, *18*, 5647

**DOI:** 10.3390/ijerph182312833

**Published:** 2021-12-06

**Authors:** Christos Livas, Konstantina Delli

**Affiliations:** 1 Dental Clinics Zwolle, 8011 CZ Zwolle, The Netherlands; 2 Department of Oral and Maxillofacial Surgery, University of Groningen, University Medical Center Groningen, 9700 RB Groningen, The Netherlands; kondelli@yahoo.gr

With great interest we have read the recently published study titled “The Implications of the COVID-19 Pandemic on the Interest in Orthodontic Treatment and Perspectives for the Future. Real-Time Surveillance Using Google Trends” authored by Sycinska-Dziarnowska et al. Using Google Trends (GT; Google, Alphabet, Mountain View, CA, USA), the authors compared search frequencies of 5 orthodontic keywords prior to the COVID-19 pandemic outbreak and at the time of pandemic, in order to measure the impact of the pandemic on the public interest in orthodontic treatment. Moreover, the popularity of “braces” vs. “invisalign” Google queries was investigated over a 5-year period [1].

At first, we would like to compliment the authors for applying behavior analytics in orthodontics, and especially in challenging times where the COVID-19 pandemic negatively affected the orthodontic treatment, and the financial and emotional wellbeing of orthodontic patients worldwide [2,3]. We hope more researchers will follow the example of the authors, and examine the infodemiology [4] of various aspects of orthodontic therapy. Analysis of online search trends may provide valuable insights into human behaviour and needs [5], and certainly deserves more attention in dental research. 

We would like, however, to express our concerns about the search strategy and the interpretation of the results. First, according to Trends Help, “search terms show matches for all terms in the query, in the language given” [6]. In other words, when entering a single word as search term in GT, search phrases containing that word will be additionally traced and all search volumes will be merged. Therefore, it is likely that the use of the word “braces” in 4 out of 5 search terms in this study might have caused overlapping of the search results. The same holds true for the search results of “invisalign”, which might have overlapped with those of “braces” terms. It can be assumed that phrases like “invisible braces”, “clear braces” or “invisalign braces”, for example, commonly used by laypersons for invisalign aligners, generated partly the same results as “braces”, “get braces”, “get braces off”, and “braces pain”. As a consequence, the results presented in the study may not reflect reality, namely, the actual numbers of queries for the orthodontic keywords. This major limitation puts at risk the results and the implications of the study. Meticulous selection of search terms could have prevented overlap of the results, and could have allowed more meaningful comparisons.

Secondly, GT search data were retrieved globally and not at country level. Nevertheless, this approach overlooks the country-specific characteristics of the pandemic, the timing and type of control measures as well as the degree of public adherence to such restrictive policies [7]. Furthermore, the recommendations of the national dental regulatory bodies and orthodontic associations for the continuity of care provision during the pandemic crisis might have also differed from country to country. In our opinion, a cross-country analysis might have been more favorable in methodological terms having produced more relevant information. 

Due to the abovementioned limitations, we fear that no solid conclusions can be drawn by the current study about the global interest in orthodontic treatment during the COVID-19 pandemic crisis. More infoveillance research exploring unique search terms and multiple countries is necessary to assess orthodontic information seeking behavior on the Internet in the pandemic era. 
